# 

**Published:** 1902-12-15

**Authors:** 


### ANNOUNCEMENTS.

### INSTITUTE OF DENTAL PEDAGOGICS. TENTH ANNIVERSARY.

The tenth annual meeting of the Institute of Dental Pedagogics will be held in Chicago, December 29th, 30th and 31st, at the Palmer House.

All who are interested in Dental Education are cordially invited to attend. The following program has been arranged:

Papers:—"Teaching Operative Procedure." Dr. C. N. Johnson; "Teaching General Anatomy to Dental Students." Dr. Boland; "Teaching Electricity and its Dental uses." Dr. W. A. Price; "Teaching Embryology." Dr. I. N. Broomell; "Teaching Applied Physics." Dr. G. V. Black; "Physical Diagnosis."

A symposium on the "Management of the Teaching of Demonstrators in the Infirmary," by four professors.

Report of Committee on "Four year Curriculum."

Report of Committee on "Nomenclature."

All new teaching appliances must be submitted to Dr. Whitslar, of Cleveland, or to Dr. Patterson, of Kansas City.

Hart J. Goslee,
*President.*

W. Earl Willmott,

*Chairman of Executive Board.*

H. B. Tileston,
*Secretary and Treasurer.*

